# The phase transformation of CuInS_2_ from chalcopyrite to wurtzite

**DOI:** 10.1186/s11671-015-0800-z

**Published:** 2015-02-27

**Authors:** Bing-Bing Xie, Bin-Bin Hu, Li-Fang Jiang, Guo Li, Zu-Liang Du

**Affiliations:** Key Laboratory for Special Functional Materials of Ministry of Education, Henan University, Kaifeng, 475004 Henan People’s Republic of China

**Keywords:** Chalcopyrite, Wurtzite, pH, CuInS_2_

## Abstract

In the present work, CuInS_2_ nanoparticles have been successfully synthesized by water-bath method with deionized water as solvent and thioglycolic acid as complexing agent at 80°C. The phase transition of CuInS_2_ from chalcopyrite to wurtzite was realized by adjusting the pH value of reaction solution. The emergence of Cu_2_S in the condition of higher pH value of reaction solution led to the formation of wurtzite CuInS_2_. This facile method that controls the phase structure by adjusting the solution pH value could open a new way to synthesize other I-III-VI_2_ ternary semiconductor compounds.

**Electronic supplementary material:**

The online version of this article (doi:10.1186/s11671-015-0800-z) contains supplementary material, which is available to authorized users.

## Background

With increasing global energy consumption, the fabrication of pollution-free, low-cost, and high-efficiency photovoltaic cells has attracted successive attention in recent years. As an I-III-VI_2_ ternary semiconductor compound with a direct bandgap of 1.5 eV at room temperature, CuInS_2_ is a promising material for photovoltaic applications because of its low toxicity, high absorption coefficient, and high theoretical photovoltaic conversion efficiency (about 25% to 30%) [1-3].

In the previous studies, CuInS_2_ has been found to exist in three different crystal structures: chalcopyrite, zinc blende, and wurtzite [4-6]. Chalcopyrite CuInS_2_ is the most common existing phase at room temperature, whereas those with zinc blende and wurtzite structures are stable only at high temperatures. Different from those of chalcopyrite phase CuInS_2_, the indium and copper atoms of wurtzite CuInS_2_ are randomly distributed over the cation sites of the lattice which allows the flexibility of stoichiometry and easily tuning the Fermi energy over a wider range [1,7,8]. Due to the differences in structure, wurtzite CuInS_2_ not only exhibits different optical properties but also may present novel properties which can expand its application. Therefore, it is meaningful to develop an effective route to realize the controlled synthesis of CuInS_2_ with different phase structures. Most of the reports about controlling the phase structure of CuInS_2_ were achieved by changing the ligand species or reaction temperatures. For example, Pan et al. firstly reported the synthesis of zinc blende- and wurtzite-structured CuInS_2_ nanocrystals by changing the ligand species [9], and Sudip K et al. reported the synthesis of zinc blende- and wurtzite-structured CuInS_2_ nanocrystals by changing the reaction temperature [10]. We also noted that for solution-phase reactions, the pH value of reaction solution can affect the complexation capability of complexing agent to metal ions, and it might be used to the control the phase structure of products. Chai et al. has reported the synthesis of cubic and hexagonal phase ZnIn_2_S_4_ by adjusting the pH value of the reaction solution [11]. This method presents a simple and eco-friendly way for the controlled synthesis of ternary nanomaterials with tailored structures.

On the other hand, CuInS_2_ in nanophase is usually synthesized in harsh conditions of high temperature and high pressure using organic solvent, which inevitably makes the reaction more difficultly controlled. In this report, we demonstrate the successful synthesis of chalcopyrite phase and wurtzite phase CuInS_2_ by a simple water-bath method at relatively low temperature of 80°C under atmospheric environment. By using deionized water as the solvent and thioglycolic acid as the complexing agent, the phase transformation of CuInS_2_ from chalcopyrite phase to wurtzite phase can be achieved by simply adjusting the pH value of the reaction solution as well as the annealing temperature. It has been found that CuInS_2_ gradually transformed from chalcopyrite to wurtzite with the increase of pH value, and the wurtzite CuInS_2_ gradually transformed to chalcopyrite phase with the increase of annealing temperature. To the best of our knowledge, this is the first report that controls the phase transformation of CuInS_2_ from chalcopyrite to wurtzite by adjusting the pH value of the reaction solution. This work may provide a feasible reference for the simple and easy synthesis of different phase-structured I-III-VI_2_ ternary semiconductor compounds.

## Methods

### Materials

All chemicals were used as received without further purification. Copper (II) chloride dihydrate (CuCl_2_ · 2H_2_O ≥ 99.0%), indium (III) sulfate anhydrous (In_2_(SO_4_)_3_ ≥ 99.99%), sodium sulfide nonahydrate (Na_2_S · 9H_2_O), and sodium hydroxide (2 mol/L NaOH solution) were all purchased from Tianjin Kermel Chemical Reagent Co. Ltd. (Tianjin, China). Thioglycolic acid (TGA) was obtained from Sinopharm Chemical Reagent Co. Ltd. (Shanghai, China). The water used in all experiments was obtained from a Millipore Milli-Q purification system and had a resistivity higher than 18.2 MΩ · cm. All experiments were carried out in water-bath pot under atmosphere.

### Synthesis of CuInS_2_ nanoparticles

CuInS_2_ was synthesized in aqueous solution via a water-bath approach. In a typical synthesis, 1 mmol CuCl_2_ and 0.5 mmol In_2_(SO_4_)_3_ were mixed with 20 mL of deionized water, then TGA aqueous solution as reducing and complexing agent (20 mmol TGA in 10 mL of deionized water) was added into the solution under constant stirring. The mixture became milky white quickly. Then, the pH value of the mixed solution was adjusted from 1.27 to 10.3 by adding aqueous NaOH solution (2 mol/L) to check the effects of pH value on the final product. Na_2_S aqueous solution as sulfur source (2 mmol Na_2_S was dissolved in 10 mL of deionized water) was then added to the mixture. After stirring for 30 min, the reaction mixture was heated to 80°C for 48 h under atmosphere conditions. Finally, the obtained solution was cooled down to room temperature. The precipitate was separated by centrifugation and washed several times with deionized water and anhydrous ethanol then dried at 60°C for 8 h.

### Characterization

The phase and crystallographic structure of the prepared products were characterized by X-ray diffraction on a Bruker D8 Advance X-ray powder diffractometer (XRD) with Cu *Kα* radiation source (*λ* = 0.15418 nm). Scanning electron microscopy (SEM) images were acquired using a FEI Nova NanoSEM 450 scanning electron microscope (FEI, Hillsboro, OR, USA). Transmission electron microscopy (TEM) images were performed on a JEOL JEM-2010 electron microscope (JEOL, Akishima-shi, Tokyo, Japan) operating at 200 kV. X-ray photoelectron spectroscopy (XPS) analysis was performed with a Kratos Axis Ultra system using monochromatic Al Kα X-rays (1,486.6 eV). The UV-vis absorption spectra were obtained by using UV-vis Spectrometer (Perkin-Elmer, Lambda 950, Waltham, MA, USA). The simulated crystal structures and wurtzite XRD patterns of CuInS_2_ were obtained by using Diamond 3.2 programs.

## Results and discussion

**Figure 1:**
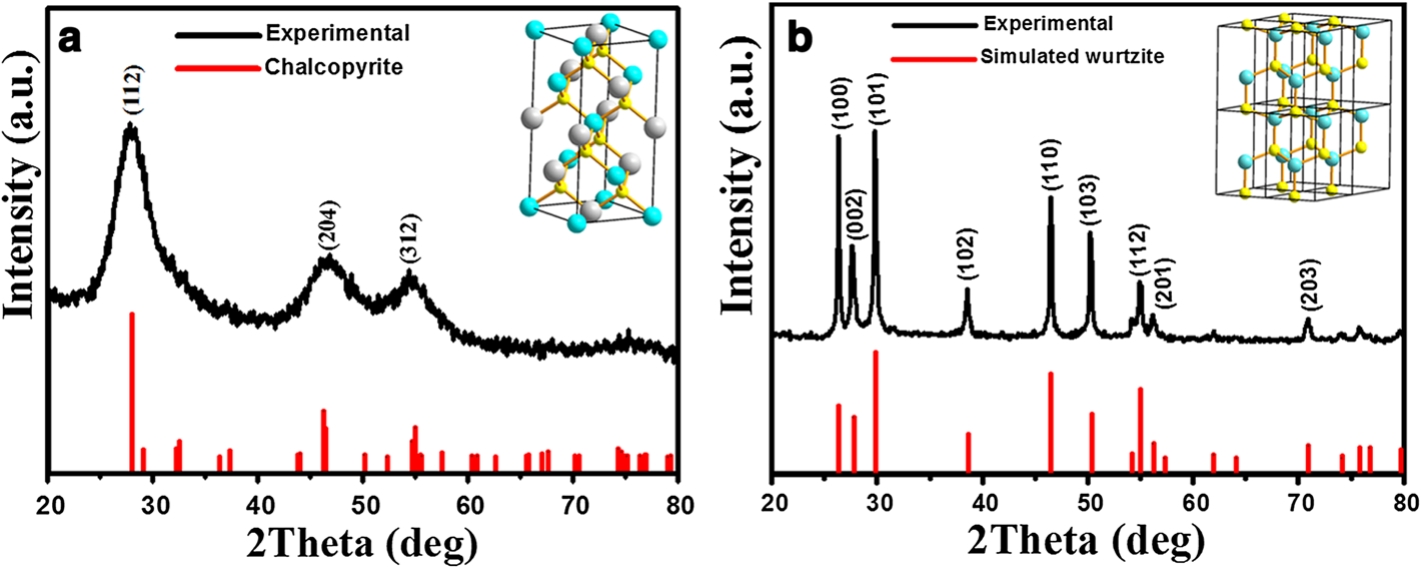
**XRD patterns of as-synthesized chalcopyrite and wurtzite CuInS**_**2**_ **. (a)** Chalcopyrite. **(b)** Wurtzite. Insets show the corresponding crystal structures.

By adjusting the pH value of the reaction solution, CuInS_2_ nanoparticles with various phase structures have been successfully synthesized at the temperature of 80°C. Figure 1a shows the XRD pattern of the products synthesized with pH value of 1.27. All the diffraction peaks could be well indexed to (112), (204), and (312) planes of the standard chalcopyrite structure of CuInS_2_ (JCPDS card file no. 85-1575), respectively. The diffraction peaks of the product are wide and weak, which indicates that the as-synthesized CuInS_2_ nanoparticles have very small sizes or poor crystallinity [12]. Figure 1b shows the XRD pattern of the products synthesized at pH of 10.3. The peak position and relative peak intensities can match well with the powder diffraction data reported for wurtzite CuInS_2_ [9,13-15]. The diffraction patterns were simulated using the lattice parameters previously reported for wurtzite CuInS_2_ (simulated by using the software Diamond 3.2, with the space group of P63mc and lattice parameters *a* = *b* = 3.897 Å, *c* = 6.441 Å [9]), and it matched well with our experimental XRD diffraction pattern. The diffraction peaks located at 2 theta of 26.3°, 27.69°, 29.75°, 38.52°, 46.4°, 50.32°, 54.94°, 56.3°, and 70.96° can be assigned to the (100), (002), (101), (102), (110), (103), (112), (201), and (203) planes, respectively. No diffraction peaks from other species can be detected, which indicates that the obtained samples are pure wurtzite CuInS_2_ without any binary sulfides of Cu_2_S, CuS, or In_2_S_3_.

**Figure 2:**
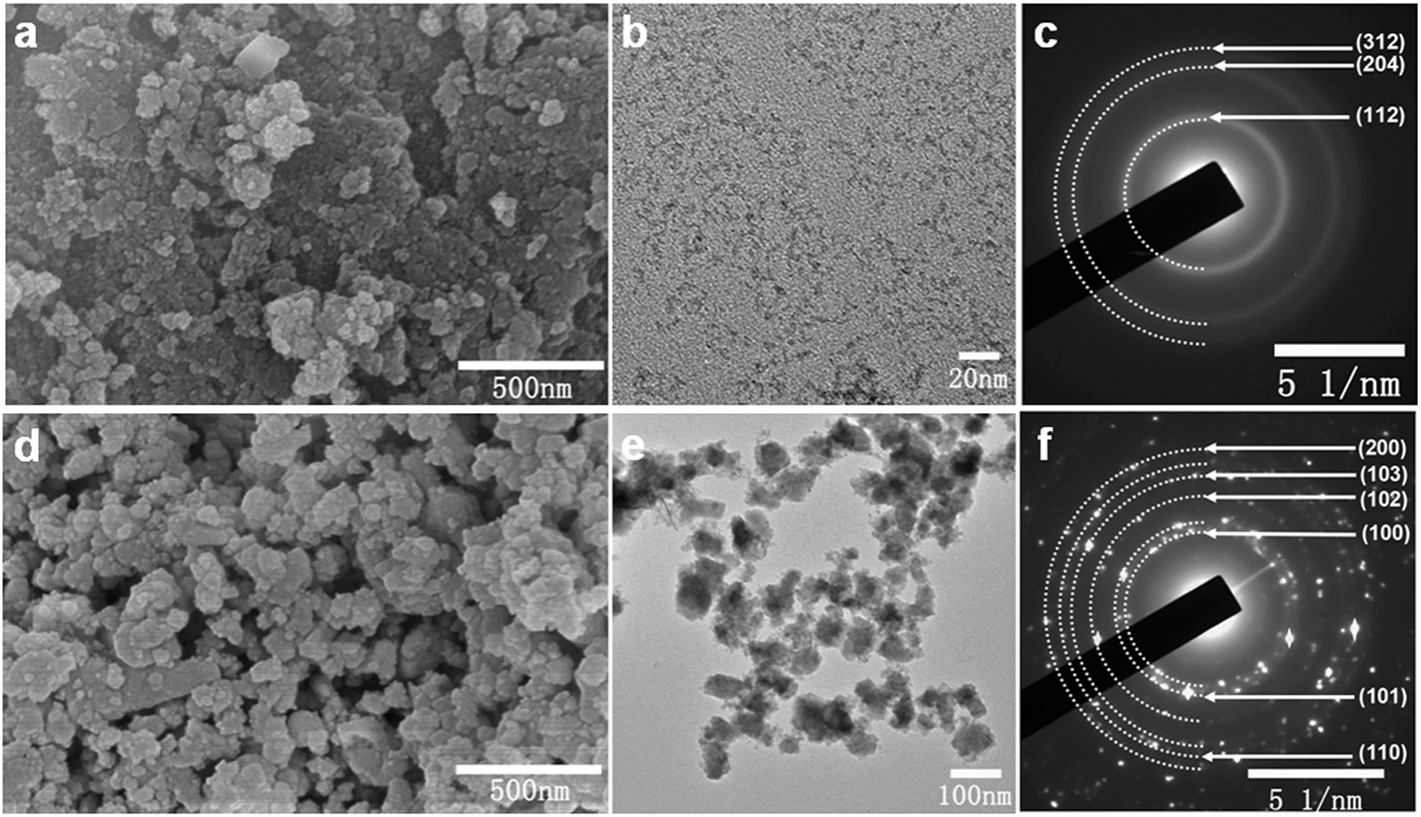
**The morphology of the chalcopyrite and wurtzite CuInS**_**2**_ **. (a, d)** SEM image, **(b, e)** TEM image, **(c, f)** SAED pattern.

The morphology of the as-synthesized CuInS_2_ was investigated by SEM, as shown in Figure 2. Figure 2a,d shows the SEM images of the as-synthesized chalcopyrite and wurtzite CuInS_2_, respectively. It reveals that the product is composed of a large quantity of nanoparticles, which are easily agglomerated due to the high active surface of nanoparticles. Further investigation was carried out by TEM. Figure 2b shows that the as-synthesized chalcopyrite CuInS_2_ has very small sizes which match well with the obtained wide and weak XRD patterns. Figure 2e shows that the as-synthesized wurtzite CuInS_2_ has an irregular feature of shape. Figure 2c,f shows the selected-area electron diffraction (SAED) of chalcopyrite and wurtzite CuInS_2_. In Figure 2c, three diffraction rings can be clearly seen, which can be well indexed as (112), (204), and (312) planes of the chalcopyrite CuInS_2_, respectively. Figure 2f shows the polycrystalline feature of the as-synthesized wurtzite CuInS_2_, according to the calculated lattice parameters based on the XRD pattern of wurtzite CuInS_2_. The diffraction rings can be well indexed to (100), (101), (102), (103), (110), and (200) planes of wurtzite phase CuInS_2_, respectively.

**Figure 3:**
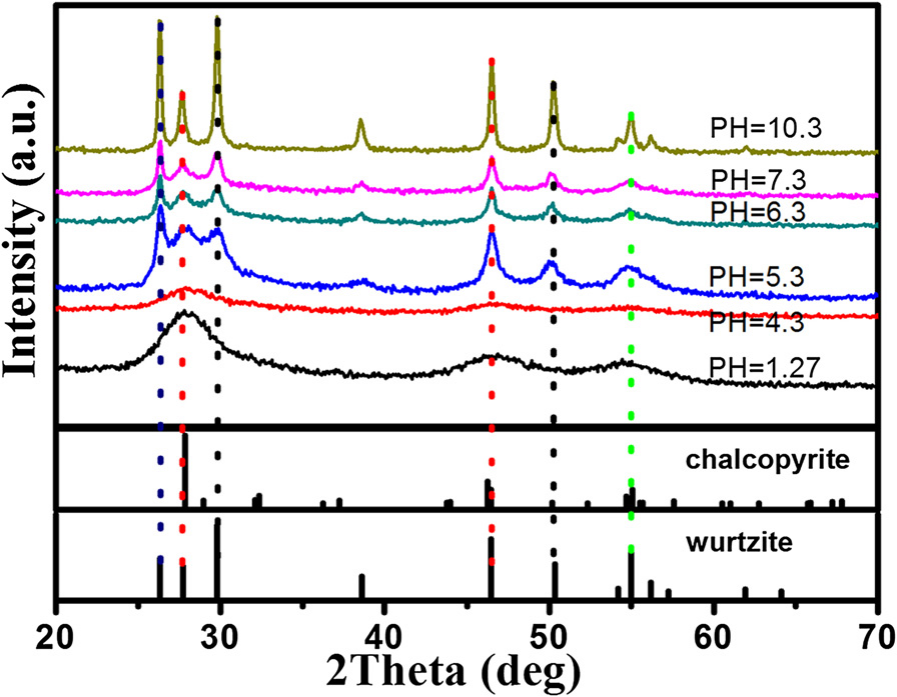
**XRD pattern of the as-synthesized CuInS**_**2**_ **nanoparticles prepared at different pH values.**

The influence of synthesis conditions on the crystal phase of products was studied by altering the pH value of reaction solution. Figure 3 shows the evolution of XRD patterns of the as-synthesized CuInS_2_ nanoparticles prepared with increasing pH values of reaction solution. It was found that the pH value of reaction solution played an important role in the determination of phase structure of the final product. As shown in Figure 3, when the pH value of the solution was 1.27, the chalcopyrite CuInS_2_ could be obtained. When the pH value of the solution increased to 5.3, the chalcopyrite CuInS_2_ transformed into wurtzite CuInS_2_. When the pH value of reaction solution is 10.3, the crystallization of the product is the best. Because of the Na_2_S shows very strong alkaline, the final reaction solution changed to alkaline solution when sufficient Na_2_S aqueous solution was added to the reaction solution with a pH value of 5.3.

According to Pearson’s Hard-Soft Acid-Base (HSAB) theory [16], a soft acid and a soft base bind more tightly than a soft base and a hard acid. Cu^+^ is a soft acid, In^3+^ is a hard acid, and the TGA is a soft base which will react preferentially with soft acid Cu^+^ [17]. The Cu-SR bond should be stronger than the In-SR bond [12,18]. Therefore, the excess of TGA can balance the reaction rate between Cu^+^ and In^3+^ and S^2−^.

From the phenomenon of the reaction process (Additional file 1: Figure S1). When the TGA is added into the mixture solution of Cu and In ions, the color of the solution changes from blue to creamy white, which indicates that CuIn(SR)_*x*_ complex is generated in the solution. If the Na_2_S was directly added into the solution without adjusting the pH value of the solution (pH = 1.27), the color of the solution will change from creamy white to orange. However, if the pH value of the solution was adjusted to alkalinity (pH = 10.3), the creamy white solution will become a colorless transparent solution. When we add the Na_2_S into the solution, the color of solution becomes gray black.

From the phenomenon of the reaction process (Additional file 1: Figure S1), It can be found that the pH value of reaction solution indeed plays an important role in the reaction. The essence is that the pH value of solution influences on the complexation of TGA. The Cu^2+^ can be quickly reduced to Cu^+^, and the CuIn(SR)_*x*_ complex (creamy white) is formed when the TGA is added into the mixed solution of Cu^2+^ and In^3+^, which makes the solution change from blue to creamy white because the Cu^2+^ is blue but Cu^+^ is colorless. As the pH value increases with the addition of NaOH, the CuIn(SR)_*x*_ complex dissociates and releases Cu^+^ into solution, and the solution changes from a creamy white to a colorless transparent solution. In this case, the Cu_2_S will be easily generated when Na_2_S is added into the solution, which makes the mixed solution change from a colorless transparent to a gray black solution. From the XRD pattern of the as-grown products of the gray black solution (Additional file 1: Figure S2), the peaks can be well indexed to (220) and (311) planes of Cu_2_S (JCPDS card file no. 02-1287), respectively, proving the formation of Cu_2_S. It is the emergence of Cu_2_S that leads to the formation of wurtzite CuInS_2_. Both Cu_2_S and wurtzite CuInS_2_ have a hexagonal structure; such a structural similarity induces the formation of wurtzite CuInS_2_ [1,19].

A series of comparative experiments have also been carried out. In any case, the pH is adjusted in the solution without TGA; the chalcopyrite nor wurtzite CuInS_2_ can be synthesized. The synthesized products both in the acidic and alkaline environment are CuS (JCPDS card file no. 06-0464) and In(OH)_3_ (JCPDS card file no. 76-1464) (Additional file 1: Figure S3). It is due to the reason that the Cu^2+^ cannot be reduced to Cu^+^ in the solution in the absence of TGA, and CuS is generated when the Na_2_S is added. Simultaneously, Na_2_S is a strong alkaline compound; as a result, In(OH)_3_ is also formed. The comparative experiment also indicates that TGA plays a crucial role for the formation of CuInS_2_ because of its complexation and reducibility [20,21].

**Figure 4:**
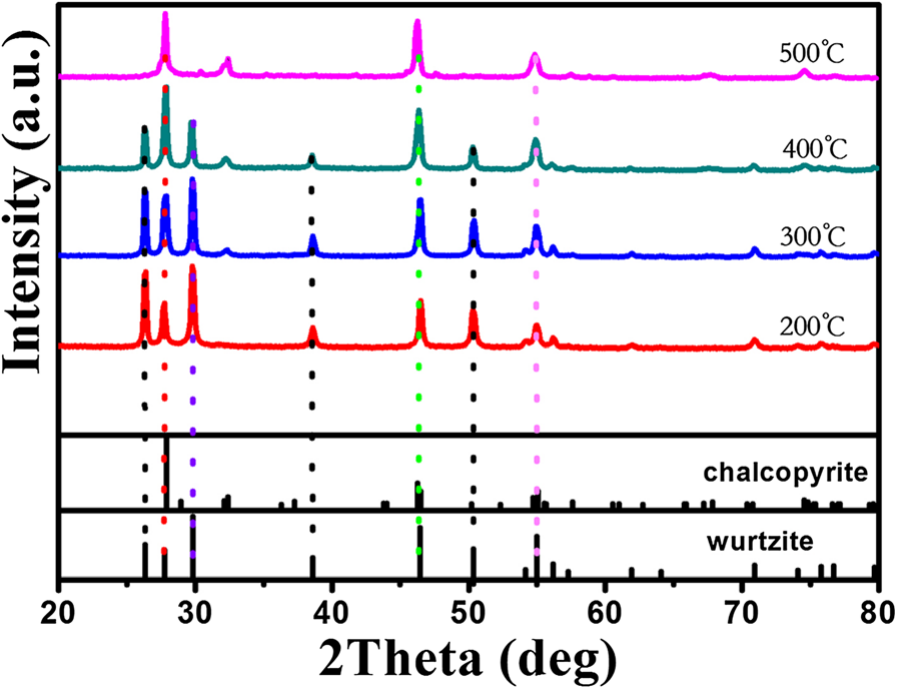
**XRD patterns of the wurtzite CuInS**_**2**_ **annealed at different temperatures.**

According to the previous report, metastable wurtzite CuInS_2_ may transform into chalcopyrite phase when wurtzite CuInS_2_ is heated to a certain temperature [6,22]. Figure 4 shows the XRD patterns of samples obtained by annealing the wurtzite phase CuInS_2_ at temperatures of 200°C, 300°C, 400°C, and 500°C, respectively. It shows that the characteristic peaks at 28° of chalcopyrite become more and more obvious with the increase of annealing temperatures. When the metastable wurtzite CuInS_2_ was annealed from 200°C to 400°C, a coexistence stage of chalcopyrite phase and wurtzite phase CuInS_2_ might exist. When the temperature reached 500°C, the wurtzite phase CuInS_2_ completely transformed into chalcopyrite phase.

**Figure 5:**
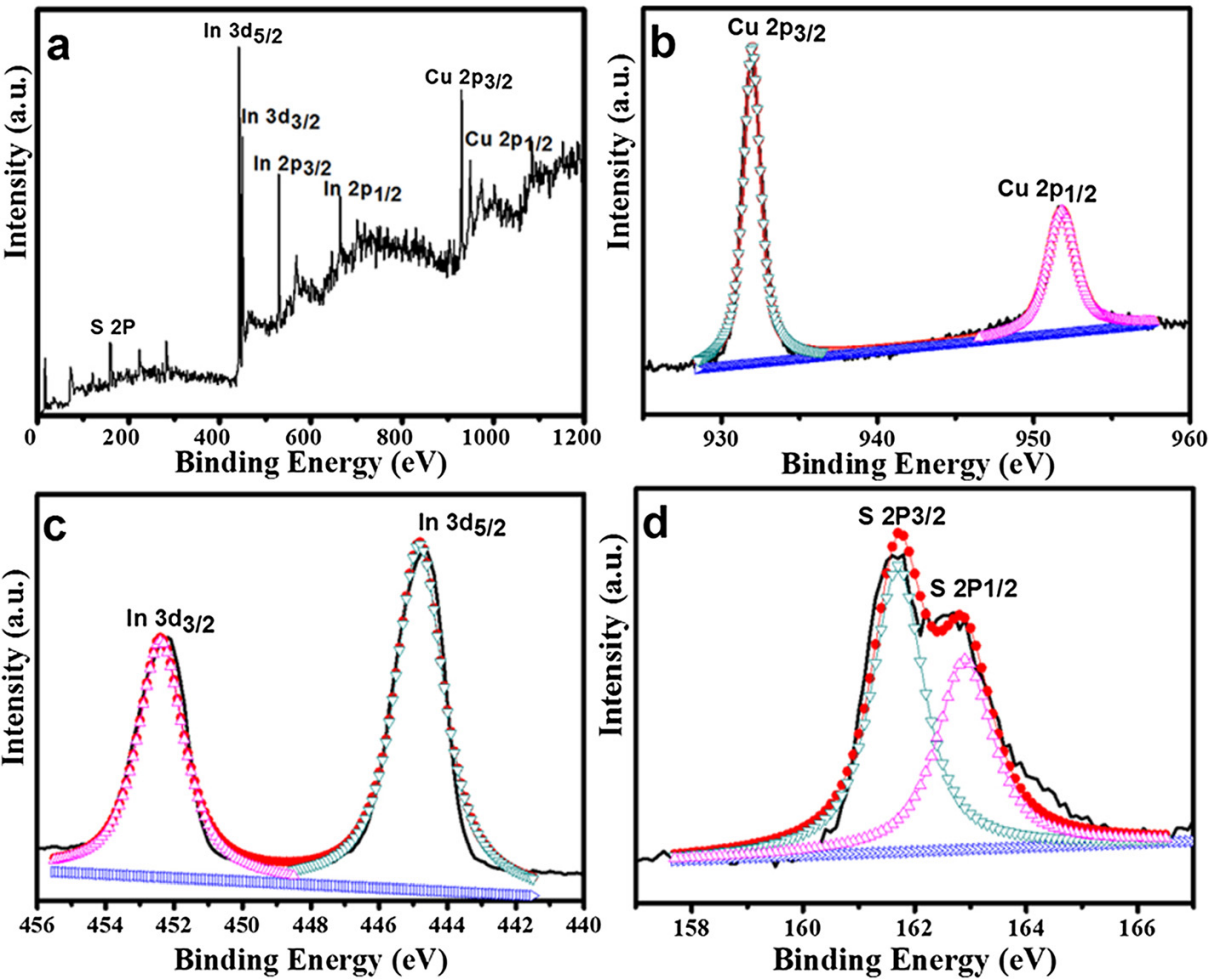
**The XPS spectra of wurtzite CuInS**_**2**_ **. (a)** Survey spectrum, **(b)** Cu 2p, **(c)** In 3d, **(d)** S 2p.

The chemical composition and valence states of wurtzite CuInS_2_ were investigated by XPS analysis. The typical survey and high-resolution spectra in regions of Cu 2p, In 3d, and S 2p are shown in Figure 5. The survey spectrum in Figure 5a indicates that the product contains Cu, In, and S elements. As shown in Figure 5b, the binding energies of Cu 2p_3/2_ and 2p_1/2_ were located at 931.9 and 951.7 eV with a peak splitting of 19.8 eV, respectively, which are in good consistence with the reported values for Cu^+^ [10,23]. In addition, the Cu 2p_3/2_ satellite peak of Cu^2+^, which is usually located at 942 eV, does not appear in the spectra [24]. Therefore, it can be concluded that the starting Cu^2+^ ions have been reduced to Cu^+^ by TGA. The In 3d_5/2_ and 3d_3/2_ peaks (Figure 5c) were located at 444.7 and 452.3 eV with a peak splitting of 7.6 eV, which matched well with that of In^3+^. The S 2p has doublet peaks of S 2p_1/2_ and 2p_3/2_ due to the spin-orbit coupling [25]. The two peaks of S 2p (Figure 5d) were located at 161.7 and 162.8 eV, respectively, with a peak splitting of 1.1 eV, which can be assigned to S^2+^. No obvious impurities could be detected in the sample.

**Figure 6:**
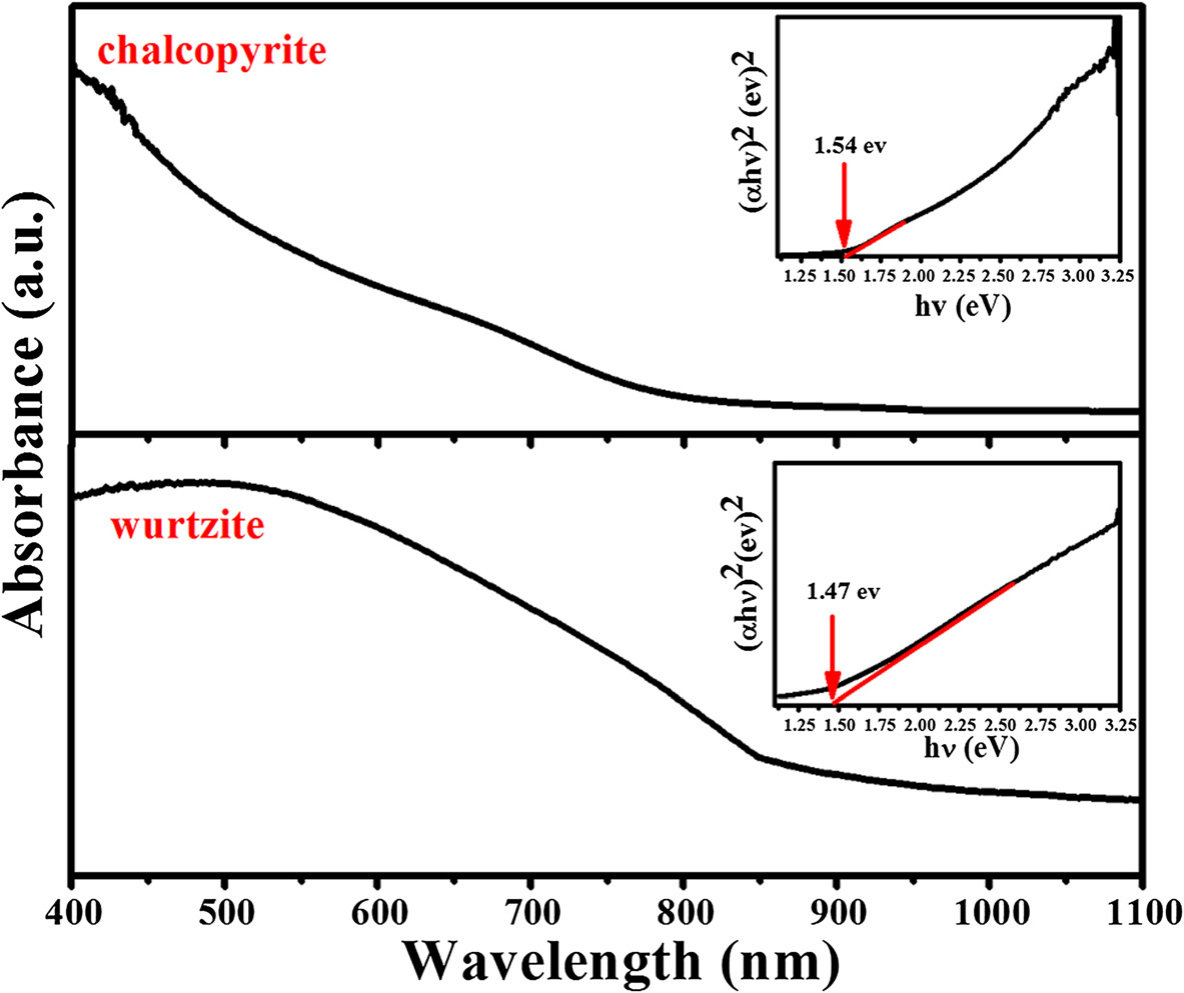
**The UV-vis absorption spectrum of chalcopyrite and wurtzite CuInS**_**2**_ **.** The insets show the bandgaps of the CuInS_2_.

Figure 6 shows the UV-vis absorption spectrum of the as-prepared chalcopyrite and wurtzite CuInS_2_ measured at room temperature. Both the two phases of CuInS_2_ show a broad and strong absorption in the visible region. Compared with chalcopyrite CuInS_2_, the wurtzite CuInS_2_ showed a higher and broader absorption in the entire visible region and near-infrared region. The bandgap can be determined by plotting (*αhν*)^2^ versus *hν* (α = absorbance, *h* = Planck’s constant, and *ν* = frequency) [26,27]. As shown in the inset picture, the calculated optical bandgap for chalcopyrite and wurtzite CuInS_2_ is about 1.54 and 1.47 eV, respectively, which is close to the bulk energy bandgap of CuInS_2_.

## Conclusions

In summary, CuInS_2_ in chalcopyrite and wurtzite phases has been successfully synthesized via a low-cost, facile water-bath method. The phase structure of as-synthesized CuInS_2_ can be easily controlled by adjusting the pH value of the reaction solution. Low-cost thioglycolic acid plays a key role in the synthesis process of CuInS_2_. Thioglycolic acid acts not only as a stabilizer and complexing agent to balance the reaction rate among Cu^+^, In^3+^, and S^2−^ but also as a reducing agent which can reduce Cu^2+^ to Cu^+^. Compared with the traditional organic phase synthesis route, this method provides a feasible way that is much simpler, greener, and cheaper, in addition to the easy control of phase structure for the mass production of CuInS_2_.

## Additional file

Additional file 1: Figure S1. The phenomenon of the reaction process. **Figure S2.** XRD pattern of the as-grown products of gray black solution. **Figure S3.** XRD pattern of the products synthesized without thioglycolic acid.
