# Supplementary material for: The phase transformation of CuInS2 from chalcopyrite to wurtzite
Source: Nanoscale Res Lett. 2015 Feb 27;10:86. doi: 10.1186/s11671-015-0800-z (PMC4385122; doi:10.1186/s11671-015-0800-z)
Supplement: Additional file 1: Figure S1. — The phenomenon of the reaction process. Figure S2. XRD pattern of the as-grown products of gray black solution. Figure S3. XRD pattern of the products synthesized without thioglycolic acid. [file 11671_2015_800_MOESM1_ESM.docx]

Supporting Information


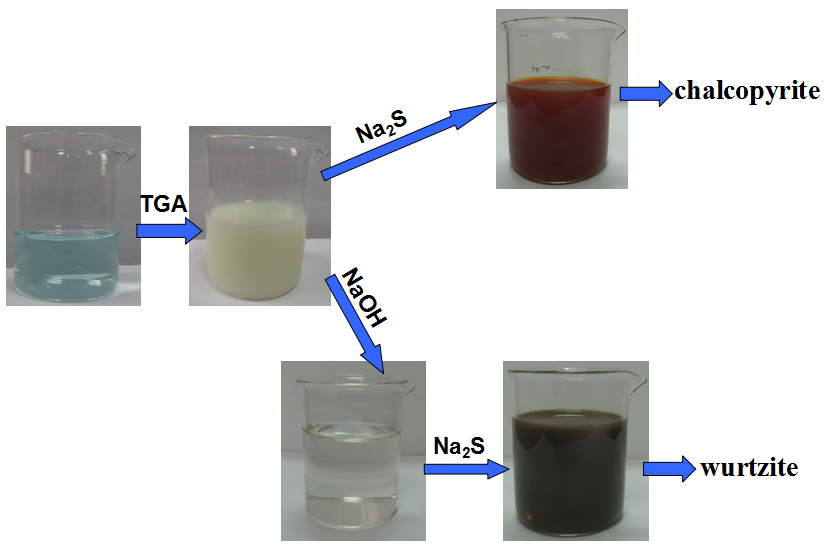


Figure S1. The phenomenon of the reaction process.


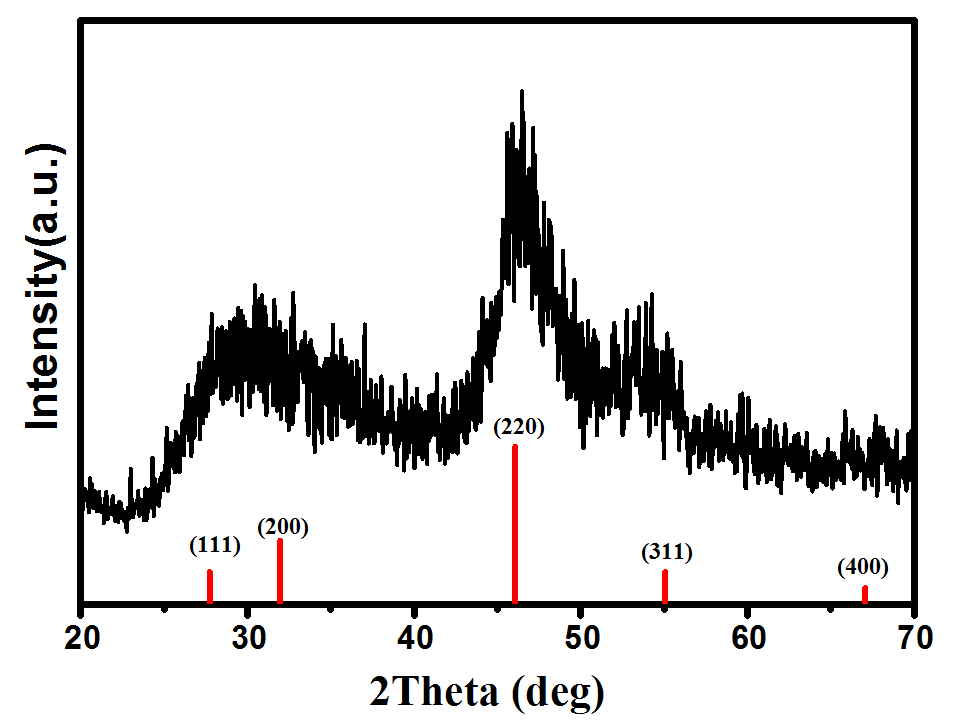


Figure S2. XRD pattern of the as-grown products of gray-black solution.


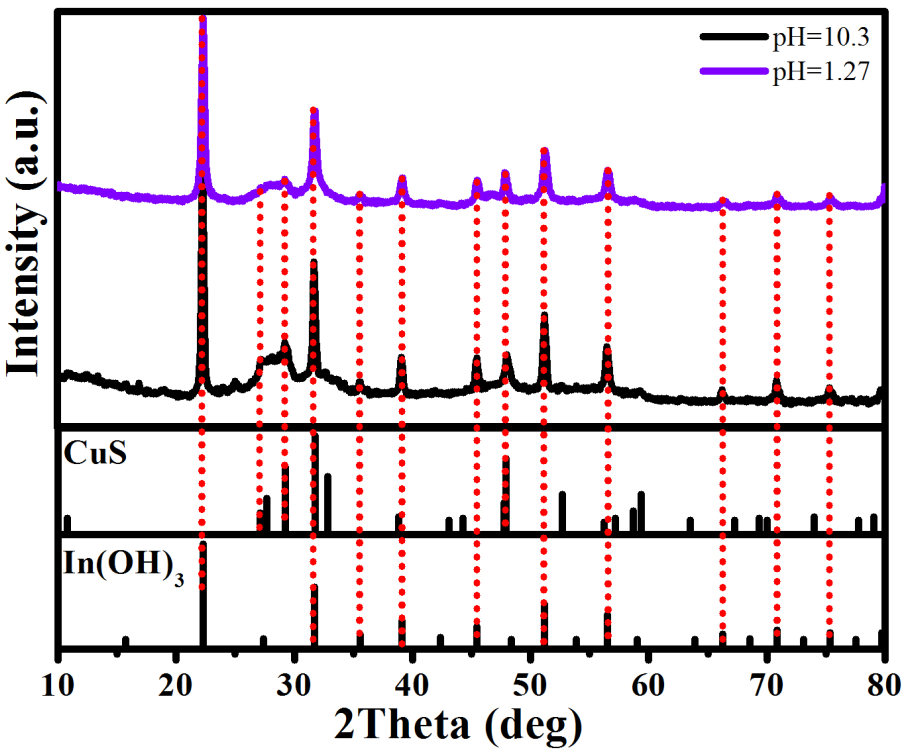


Figure S3. XRD pattern of the products synthesized without thioglycolic acid.
